# Absolute quantitation of binding antibodies from clinical samples

**DOI:** 10.1038/s41541-023-00793-w

**Published:** 2024-01-06

**Authors:** Chan Tang, Annemiek Verwilligen, Jerald Sadoff, Boerries Brandenburg, Eveline Sneekes-Vriese, Tom van den Kerkhof, Lieve Dillen, Lucy Rutten, Jarek Juraszek, Katleen Callewaert, Sarah Janssen, Jeroen Huizingh, Zelda Euler, Tom Schilperoord, Marc Verhemeldonck, Johannes P. M. Langedijk, Jenny Hendriks, Daniel J. Stieh

**Affiliations:** 1 Janssen Vaccines & Prevention, Leiden, The Netherlands; 2 Janssen Research & Development, Beerse, Belgium; 3 Present Address: Vaccine Company, Inc., South San Francisco, CA USA

**Keywords:** Antibodies, Vaccines, Vaccines

## Abstract

The quantitation of antibody responses is a critical requirement for the successful development of vaccines and therapeutics that often relies on the use of standardized reference materials to determine relative quantities within biological samples. The validity of comparing responses across assays using arbitrarily defined reference values is therefore limited. We developed a generalizable method known as *MASCALE* (*Ma*ss *S*pectrometry Enabled *C*onversion to *A*bsolute *L*evels of *E*LISA Antibodies) for absolute quantitation of antibodies by calibrating ELISA reference sera using mass spectrometry. Levels of proteotypic peptides served as a proxy for human IgG, allowing the conversion of responses from arbitrary values to absolute amounts. Applications include comparison of binding assays at two separate laboratories and evaluation of cross-clade magnitude-breadth responses induced by an investigational HIV-1 vaccine regimen. *MASCALE* addresses current challenges in the interpretation of immune responses in clinical trials and expands current options available to make suitable comparisons across different settings.

## Introduction

Traditional methods for the characterization and quantitation of antigen-specific antibodies in biological samples utilized multiple techniques. Historically, these techniques included combining sera dilutions with polysaccharide antigens, resulting in precipitates that were analyzed by Kjeldahl nitrogen reactions to accurately quantitate the antibody–protein complex^1,2^. Electrophoresis combined with densitometric scanning, single radial immunodiffusion, laser nephelometry and turbidity assays followed, relying on the biophysical properties of antibody–antigen interactions to isolate and quantify immune complexes^3–6^. The quantity of antibodies is generally obtained by referencing a standard of known concentration. Despite major technological advances, these methods were labor-intensive, often inconsistent, and lacking in precision and overall sensitivity for quantitation of serum immunoglobulins^7^. Mass spectrometric techniques in combination with liquid chromatography were later introduced to separate, characterize, and quantify ionized particles within a sample by mass-to-charge ratio. This approach has more recently been used to measure levels of human monoclonal antibodies in animal models. Direct quantitation of antigen-specific antibody subclass and subtypes by mass spectrometry has been employed to measure biomarker levels in response to infection and vaccination but has not been broadly adopted as a general method to evaluate immune responses^8,9^.

To achieve higher throughput antigen-specific ligand binding assays, measurement of antibody levels was performed with techniques based on the enzyme-linked immunosorbent assay (ELISA). Current methods for quantitation of binding antibodies in clinical samples utilize polyclonal serum or purified antibodies as a reference standard, where an absolute level of specific antibody is unknown. Regulatory guidance for assay development and clinical evaluation recommends matching the reference material matrix to the sample type being analyzed, making polyclonal serum reference standards the appropriate choice for evaluation of clinical sera^10,11^. Variation in binding of reference sera across assay formats with even subtle underlying differences in methodology limits comparisons and complicates interpretation of results. Arbitrarily defined reference values mean that quantitative comparison is not possible, and information on absolute magnitude and response breadth across different assays cannot be obtained. Protective levels of immunity for a given pathogen can only be related from one assay to another with specific assumptions. Using known amounts of purified antibodies as a reference standard can facilitate absolute quantitation, but the availability of such reagents relies on efficient capture and purification from biological matrices. Successful purification requires stable immobilization of biological agents onto appropriate supports without altering the binding properties of the ligand^12^. To overcome difficulties in the availability of sample-purified antibodies, monoclonal antibodies can be considered for quantitation. However, variation in binding profiles based on antibody specificity mean results cannot be fairly compared across diverse antigens and their relevance for use as generalized reference standards is restricted.

Methods for absolute quantitation of antibodies from clinical samples across a diverse range of antigenic targets are thus far limited. The establishment of such methods would allow comparison of immune responses across relevant classes of antigens, between laboratories, and aid in defining assay-independent benchmarks for clinical benefit, foregoing the need for use of international standard panels. Here we describe the application of a novel approach for absolute quantitation of binding antibodies through mass spectrometric calibration of ELISA responses to the amounts of 2 proteotypic peptides used as surrogates for human IgG. This approach, termed *MASCALE* (Mass Spectrometry Enabled Conversion to Absolute Levels of **E**LISA Antibodies), is broadly applicable for the evaluation of humoral immune responses across diverse disease areas.

## Results

### *MASCALE* methodology: conversion formula generation from mass spectrometric calibration of ELISA reference standard

**Fig. 1:**
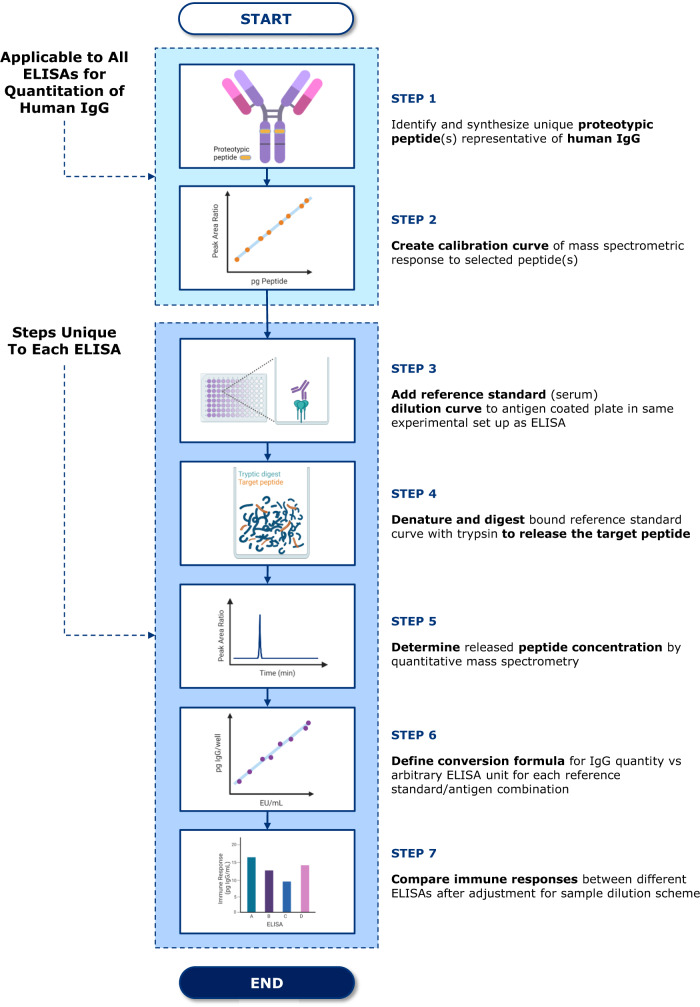
Mass spectrometry enabled conversion to absolute levels of ELISA antibodies—MASCALE—method outline. Step 1: Identification and synthesis of proteotypic peptide(s) representative of human IgG. Peptides selected are unique to human IgG, have favorable mass spectrometry characteristics and are detectable from tryptic digests. Step 2: Construction of calibration curve(s) by mass spectrometry for peptide(s) selected in Step 1. Peptide calibration curves establish the linear relationship between peptide quantity and mass spectrometric signal expressed as peak area ratio. Steps 1 and 2 apply to all ELISAs where human IgG is to be quantified via the *MASCALE* method. Step 3: Binding of ELISA reference standard samples to coated antigen. The binding of the reference standard dilution curve to coated antigen is performed using the same experimental procedure and format as done for sample analysis, followed by a wash step to remove unbound antibodies and other serum components. Step 4: Sample denaturation and tryptic digestion. Antigen-antibody complexes are denatured and digested releasing target peptide(s). Step 5: Quantitation of target peptide(s) by mass spectrometry. Tryptic digests are subjected to targeted quantitative mass spectrometry and peptide quantity in the sample is determined using the peptide calibration curve generated in Step 2. Step 6: Generation of *MASCALE* conversion formula. The linear relationship between total log_10_ IgG (peptide) quantity per ELISA well and assigned log_10_ arbitrary unit is established. Step 7: Comparison of immune responses. Reportable values generated from samples assessed in the ELISA are converted from concentrations in arbitrary units to absolute units after applying the corresponding conversion formula from Step 6 and adjusting for sample dilution scheme of the respective ELISA.

**Fig. 2:**
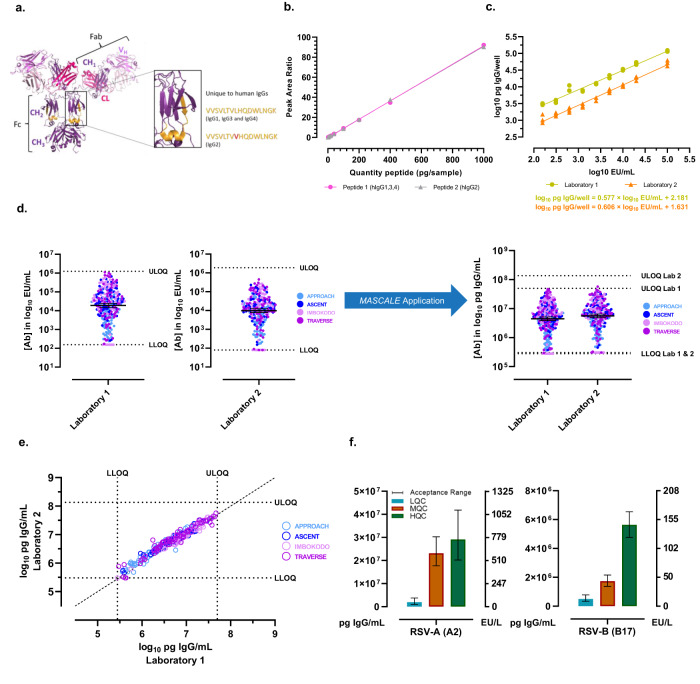
*MASCALE* method implementation for cross-laboratory assay transfer and cross-antigen comparisons. **a** Unique peptide representative of human IgG1, IgG3, and IgG4 highlighted in the CH2 domain (orange) on the structure of human IgG1 (PDB ID: 1HZH). Peptides unique to human IgG1, IgG3, and IgG4 (VVSVLTVLHQDWLNGK) and human IgG2 (VVSVLTVVHQDWLNGK) are used to quantify total bound IgG in the reference standard samples. **b** Peptide calibration by mass spectrometry. Target peptide calibration curve for peptide VVSVLTVLHQDWLNGK (pink) representative of human IgG1, IgG3, and IgG4 and peptide VVSVLTVVHQDWLNGK representative of human IgG2 (gray) are generated for downstream quantitation of antigen-specific binding antibodies in ELISA performed in Laboratory 1 demonstrating relationship between peptide quantity and mass spectrometric readout in peak area ratio (light/heavy isotope). **c** Relationship between total antigen-specific IgG concentration in ELISA reference standard samples and assigned arbitrary values in EU/mL. Conversion formulas are generated for ELISAs performed at Laboratory 1 (green) and Laboratory 2 (orange) by linear regression to obtain slope and intercept values. **d** Cross-laboratory comparison of clade C (C97ZA)-specific ELISA responses in arbitrary (EU/mL; left) and absolute (pg IgG/mL; right) units following conversion of data using the *MASCALE* method. Results from 4 clinical studies (APPROACH: light blue, ASCENT: dark blue, IMBOKODO: light pink, TRAVERSE: purple) can be appropriately compared across laboratories once immune responses are converted into absolute values. Bars represent geometric mean and 95% confidence interval. Dotted lines represent the lower limit of quantification (LLOQ) or upper limit of quantification (ULOQ) for the respective assay. **e** Cross-laboratory correlation of ELISA responses from 4 clinical studies (A004: light blue, ASCENT: dark blue, IMBOKODO: light pink, TRAVERSE: purple) in absolute units (log_10_ pg IgG/mL). Dotted lines represent the lower limit of quantification (LLOQ) or upper limit of quantification (ULOQ) for the respective assay. Solid line represents the unit line. **f** Low (LQC: blue), Medium (MQC: brown) and High (HQC: green) Quality Control sample responses quantified in absolute units (pg IgG/mL, left axis) for RSV subtype A (A2) and B (B17) prefusion F ELISAs. The corresponding arbitrary response in EU/L is shown on the right axis. The mean result from >60 replicates across multiple runs is shown. Bars represent the assay acceptance criteria, calculated as the 95% β-expectation tolerance intervals for the QC sample.

To meet the need for absolute measurements to facilitate cross-assay comparisons of binding antibody responses assessed by ELISA, we developed the *MASCALE* methodology. The approach follows a multi-step process (Fig. 1), starting with the identification and synthesis of proteotypic peptides representative of human IgG (Fig. 1, Step 1). Peptides used as a surrogate for human IgG1, 3, and 4, as well as human IgG2 (Fig. 2a and Supplementary Fig. 1), were selected for mass quantitation from tryptic digests. Peptides were selected based on several key attributes, including reliable release following tryptic digestion, favorable mass spectrometry characteristics, and being uniquely present among human IgG proteins^13^. To measure IgG1, 3, and 4, the sequence VVSVLTVLHQDWLNGK was selected, while IgG2 was quantified using VVSVLTVVHQDWLNGK; the amount summed represented total human IgG. Peptide calibration curves were created using targeted quantitative mass spectrometry for further downstream processing of ELISA responses, establishing the linear relationship between peak area ratio and peptide concentration (Fig. 1, Step 2). The peptide calibration procedure is applicable across all assays for quantitation of human IgG binding antibody responses across the assay range. Subsequent steps require measuring the binding of reference standard samples to antigen for each ELISA selected for quantitation of responses. To convert antibody concentrations for the reference standard curve to the mass of the target surrogate peptides, the ELISA was partially recapitulated by performing target antigen coating and binding of reference standard samples using the same methodology as applied for clinical sample analysis (Fig. 1, Step 3). Instead of completing the ELISA by detecting the bound antibodies via a secondary antibody, samples were subjected to preparative steps for mass spectrometric analysis. Bound proteins were denatured and digested with rLys-C/trypsin and processed by filter-assisted sample preparation and solid-phase extraction (Fig. 1, Step 4). The peak area signals of the target peptides liberated were then obtained by mass spectrometry (Fig. 1, Step 5). Using the peptide calibration curve, IgG amounts were deduced, and a conversion formula was defined to relate arbitrary ELISA units (EU)/mL assigned to the reference standard curve samples to the quantity of IgG measured (Fig. 1, Step 6). Next, we applied the conversion formula to ELISA data generated in the corresponding assay to express responses in the amount of IgG per mL serum (Fig. 1, Step 7). The conversion formula is valid for application to all subsequent data generated without the need for repetition of the procedure, so long as the ELISA remains unchanged.

### Implementation of *MASCALE* for cross-laboratory and cross-antigen comparisons

The *MASCALE* method was qualified by evaluating the parameters outlined in Supplementary Table 1. The approach was subsequently implemented to enable a comparability assessment between two laboratories, each performing a validated reference standard-based ELISA measuring total IgG responses to a Human Immunodeficiency Virus (HIV-1) envelope (Env) antigen (Supplementary Tables 2 and 3). The methods used the same serum reference standard but different sample dilution scheme, blocking buffer composition, and source for antigen production, including small differences in sequence. Target peptide calibration curves were generated (Fig. 2b and Supplementary Table 4) and conversion formulas for absolute quantitation of the reference standards were defined (Fig. 2c). When comparing responses in EU/mL, arbitrary readouts from Laboratory 1 measured greater numerical levels compared to Laboratory 2 (Fig. 2d, left), with a geometric mean concentration of 19,010 EU/mL compared to 9873 EU/mL, respectively. While the proportionality criterion was met (90% CI of the slope within [0.80, 1.25]), a systematic difference was observed beyond the set equivalence limits routinely used for comparability testing within the same assay (90% CI within [−0.155 log_10_; 0.155 log_10_]) (Supplementary Fig. 2). By applying the *MASCALE* conversion formula for absolute quantitation and correcting for assay dilution steps, concordance was observed between Laboratory 1 and Laboratory 2, with consistent antibody levels achieved between laboratories for the same samples from 4 independent clinical studies (Fig. 2d, right, e). Results met acceptance criteria for proportionality and systematic difference evaluation, with a slope of 0.96 (90% CI: 0.94–0.98), and an average difference in log_10_ pg IgG/mL across samples of 0.102 log_10_ (90% CI: 0.090–0.113) (Supplementary Fig. 2). Concordance testing outcomes allowed cross-laboratory comparisons, supporting transfer of clinical sample analysis between laboratories.

The method can also be applied to assess binding antibody responses across antigens. Variation in the circulating strains of many viruses makes understanding the levels of pre-existing or induced immunity to each strain important. Two predominant subtypes of Respiratory Syncytial Virus (RSV) are circulating globally. Genetic drift in the fusion protein and changes in the predominant circulating strain each season complicate efforts for effective interventions since efficacy and evaluation of immune responses against all relevant strains is needed^14^. To prepare for concurrent assessment of pre-existing binding antibody responses and those elicited by vaccination, ELISAs for RSV-A (Strain A2) and RSV-B (Strain B17) prefusion F protein were established^15^. To enable clinical sample analysis, the quality control (QC) samples used to determine assay validity and monitor trending were tested multiple times across runs to establish their acceptance ranges for each assay. Implementing the *MASCALE* method allowed comparison of responses for assay controls and their acceptance ranges using the same absolute metric, even while the assays used different sources of reference standard and QC samples (Fig. 2f). The assay-specific conversion formulas can be subsequently applied to all clinical data.

### HIV-1 Env antigen panel selected for sequence diversity in clades B and C shows distinct binding profiles when characterized with polyclonal sera and monoclonal antibodies

To determine vaccine coverage for diverse variants of a pathogen, such as Influenza, SARS-CoV-2, or HIV-1, measurement of immune responses across a larger representative set of distinct strains is essential^16,17^. We further applied *MASCALE* for evaluation of immune responses induced by the Ad26-based HIV-1 vaccine under development. Since efficacy studies are conducted in regions with predominance for a single HIV-1 clade, determining the cross-clade coverage of a vaccine regimen represents a major challenge for the field. Establishing comparative cross-clade immune responses for a biomarker correlating with protection could circumvent the need for extensive studies in multiple regions, as immune responses to relevant clades could be effectively bridged, and efficacy against untested clades extrapolated.

**Fig. 3:**
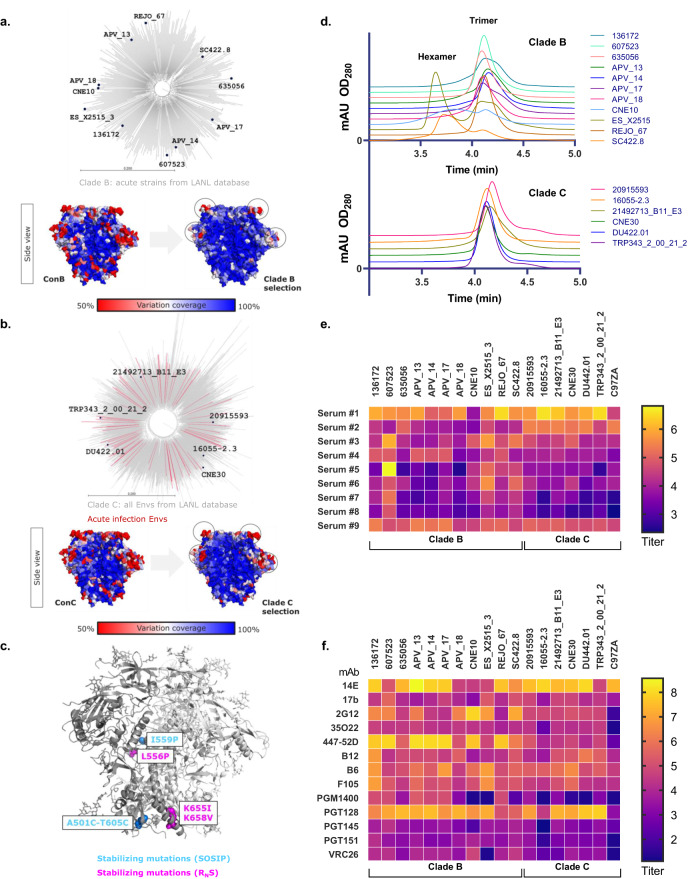
Selection and characterization of representative acute HIV-1 Env antigen panel selected for sequence diversity in clade B and C. **a** Selection of clade B strains shown on phylogenetic tree of acute clade B sequences available in the Los Alamos National Laboratory (LANL) database (top). Amino acid coverage of the selected clade B strains modeled on the crystal structure of HIV-1 Env relative to the consensus clade B sequence (bottom). Circles represent parts of the hypervariable loop visible in the crystal structure. **b** Selection of clade C strains shown on phylogenetic tree of all clade C sequences available in the LANL database (top). Strains were selected from a panel of acute clade C viruses in Southern Africa (Rademeyer panel; red) and included in addition reference strains with acceptable expression profiles. Amino acid coverage of the selected clade C strains modeled on the crystal structure of HIV-1 Env is compared to the consensus clade C sequence (bottom). Circles represent parts of the hypervariable loop visible in the crystal structure. Scale bar corresponds to 0.200 proportion amino acid difference. **c** Structure-based stabilizing mutations highlighted on the crystal structure of the HIV-1 Env protein. SOSIP mutations are highlighted in blue. Stabilizations used in combination with consensus repair methodology (R_N_S) are shown in pink. Scale bar corresponds to 0.200 proportion amino acid difference. **d** Size-exclusion chromatography profiles for HIV-1 Env clade B and clade C antigen panel. Purified proteins show largely uniform trimeric content, with occasional hexamer contributions for some clade B strains. **e** Heatmap showing polyclonal serum binding responses to HIV-1 Env antigen panel assessed by ELISA. Antigens were characterized using a panel of commercially sourced sera from HIV-1-infected individuals. Average log_10_ endpoint titers were plotted. **f** Heatmap showing monoclonal antibody binding responses to HIV-1 Env antigen panel assessed by ELISA. Antigens were characterized using a panel of HIV-1 Env-specific monoclonal antibodies. Average log_10_ endpoint titers were plotted.

To advance our understanding of the cross-clade coverage of antibody responses induced by the HIV-1 vaccine regimen, we established a panel of 17 HIV-1 Envs from clades B and C to measure the magnitude and breadth of the immune response. Strains were selected for representative diversity and antigenic properties characteristic of the clade as a whole (Fig. 3a, b). Selected strains had loop length and charge distribution representative of their corresponding clades. Similarly, glycosylation sites showed the diversity expected from the two clades (Supplementary Figs. 3 and 4). The antigen panel achieved a high degree of amino acid coverage throughout the Env sequence, with >90% of variation at every independent position represented within the antigen set (Fig. 3a, b). The selected antigen panel was engineered to include a minimal number of consensus repair and structure-based stabilization mutations (Fig. 3c and Supplementary Fig. 5) to ensure expression and consistent purification of trimeric proteins in native, prefusion conformation, while preserving the diversity of viral attributes and epitope integrity^18,19^. Proteins were produced and purified under the same conditions, resulting in preparations largely uniform in trimer content, with occasional hexamer contributions for some strains (Fig. 3d). We further characterized the panel with commercially sourced polyclonal sera from HIV-1-infected individuals (Fig. 3e) and a set of monoclonal antibodies specific for HIV-1 Env (Fig. 3f). Binding profiles revealed distinct antigenic patterns that were not conserved across antigens for either the polyclonal serum elicited by natural infection or from monoclonal antibodies with different antigenic specificities. The broad variation across antigens highlights the diversity in the panel as well as the limitations of using standard reference panels to enable meaningful comparisons between ELISAs with related antigens. The *MASCALE* method quantifies binding antibody responses for the reference standard of each assay, establishing a quantitative link to the assigned reference values and allowing downstream conversion of immune responses across the panel of antigens to the same absolute unit.

### Application of *MASCALE* allowed determination of magnitude-breadth responses to a panel of clade B and C HIV-1 Env antigens for a HIV-1 vaccine clinical study

**Fig. 4:**
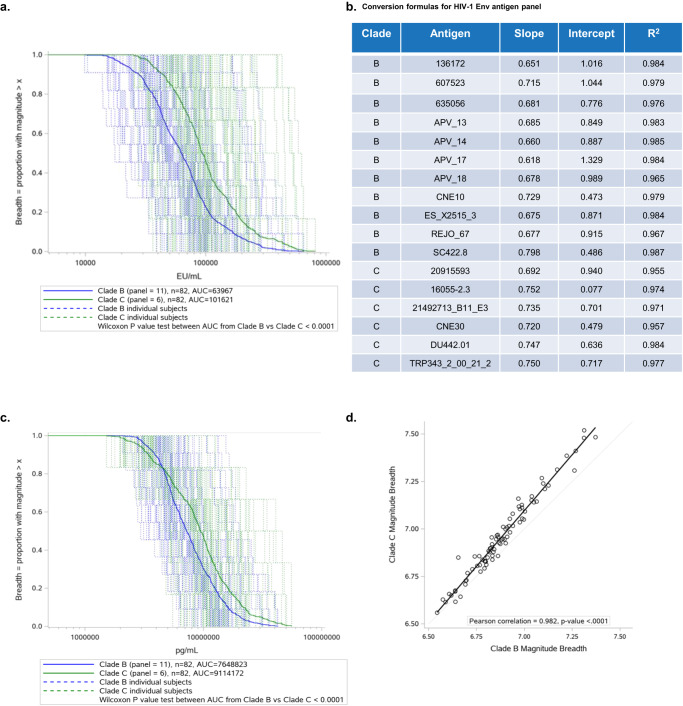
Application of *MASCALE* method for determination of magnitude-breadth responses from ASCENT to a panel of representative clade B and clade C HIV-1 Env antigens. **a** Magnitude-breadth plot for clade B and clade C arbitrary EU/mL responses for participants from the ASCENT HIV-1 vaccine clinical study assessed at peak immunogenicity (week 52). Individual and population-level (bold) clade B and clade C responses based on the average magnitude-breadth across all participants evaluated are shown (clade B: blue; clade C: green). A Wilcoxon log-rank test was applied to the area under the curve (AUC) for clade B and clade C and the resultant *P* value is shown. **b** Conversion formulas showing relationship between EU/mL arbitrary values and pg IgG/well absolute values for the reference standard of each ELISA in the HIV-1 Env antigen panel. Formulas were derived by linear regression showing slope, intercept and *R*^2^ values. **c** Magnitude-breadth plot for clade B and clade C absolute pg IgG/mL responses for participants from the ASCENT HIV-1 vaccine clinical study assessed at peak immunogenicity (week 52). Individual and population-level (bold) clade B and clade C responses based on the average magnitude breadth across all participants evaluated are shown (clade B: blue; clade C: green). A Wilcoxon log-rank test was applied to the area under the curve (AUC) for clade B and clade C and the resultant *P* value is shown. **d** Correlation between clade B and clade C magnitude-breadth responses per participant assessed with the HIV-1 Env ELISA panel. The associated Pearson correlation coefficient is shown.

ELISAs for the 17 antigen HIV-1 Env panel were qualified prior to analysis of samples from the Phase 1/2a ASCENT clinical study (Supplementary Table 5)^20^. All assays had a percent coefficient of variation less than 30%, with medians of 10.3% and 8.7% for the clade B and C panel, respectively. Assays were developed using parallel methodologies for antigen production, coating, reference sera, and detection, to enable successful interpretation of immune response breadth for two distinct viral clades. The same regimen being tested for efficacy was selected for the assessment of vaccine-induced cross-clade immune responses at peak immunogenicity. Magnitude-breadth curves were constructed to assess participant responses to the entire antigen panel and evaluate differences in clade B and C directed responses. The proportion of vaccinees with an immune response greater than a given concentration is shown separately for each clade. When performing analysis using arbitrary outputs in EU/mL as source data, results indicate a statistically significant 1.59-fold (95% CI: 1.54–1.64, *P* < 0.0001) difference between population-level magnitude-breadth responses expressed as area under the curve (AUC) for clade B and clade C (AUC 63,967 vs 101,621, Fig. 4a).

Deriving the *MASCALE* conversion formula for each assay showed good model fit and a strong linear relationship between the log_10_ EU/mL and log_10_ pg IgG per well (pg IgG/well) values, with *R*^2^ > 0.95 (Fig. 4c). Minimal variation was seen in the value of the slope (ranging from 0.618 to 0.798), whereas more spread was observed in the intercept (ranging from 0.077 to 1.329) (Fig. 4b). EU/mL reportable values obtained from each ELISA were translated to pg IgG per mL (pg IgG/mL) quantities using the corresponding *MASCALE* conversion formula before further data analysis. Vaccine recipients (*N* = 82) responded to all 17 antigens evaluated and showed considerable variation in individual response magnitudes and population-level geometric mean responses per antigen. Magnitude-breadth curves showed that clade C population-level responses were 1.19-fold (95% CI: 1.16–1.22) higher than clade B (AUC 9,114,172 vs 7,648,823, Fig. 4c), a smaller difference compared to when assessed in EU/mL, highlighting the qualitative differences when interpreting results using arbitrary units instead of absolute concentrations. A Wilcoxon log-rank test of AUC values confirmed statistical significance (*P* < 0.0001). There was strong correlation observed between individual participant magnitude-breadth response to clade B and clade C (Pearson correlation 0.982, *P* < 0.0001) (Fig. 4d). We confirmed that immune responses induced by the HIV-1 vaccine regimen covered antigens representative of acute circulating clade B and C strains. To further define cross-clade coverage for the vaccine, the methodology could be applied to an expanded panel of antigens, covering additional clades with global significance.

## Discussion

Evaluation of humoral immune responses using a single assay performed at the same laboratory represents the simplest way to reliably evaluate immune responses during vaccine and drug development. The ELISA is often the assay of choice for the evaluation of binding antibody responses to vaccines or other therapeutics due to its simplicity, specificity, sensitivity, and throughput. The use of independent reference standards for relative quantitation using arbitrarily set ranges limits the meaningful interpretation of data across assays, even when calibrated to an international standard^21^. Affinity capture methods to obtain purified antibodies for absolute quantitation often require large amounts of input material due to inefficiencies in capture and loss of product during purification. We describe the *MASCALE* method that facilitates data analysis and interpretation by enabling the evaluation of data converted to a single absolute metric, removing the need for use of internationally established reference panels to compare between data sets. The method exploits well-established techniques combined in a unique manner and allows quantitation of affinity-captured antibodies from polyclonal serum in situ, permitting straightforward comparisons of binding antibody concentrations in clinical samples without adapting routine sample analysis or impacting throughput. This approach also allows retrospective quantitation of historically generated data to evaluate against current data sets, provided the assays are still available. *MASCALE* should be applied for each unique ELISA to enable absolute quantitation of responses. Should changes in methodology be implemented, the conversion formula must be regenerated.

The sample preparation procedure was optimized prior to implementation. Bovine serum albumin was included during denaturation in urea to minimize adsorption of antibodies and peptides. Filter-assisted sample preparation^22^ was used for sample digestion and resulted in improved recovery of the target peptides compared to digestion in solution. Conditions were further optimized to minimize ex vivo deamidation of the target peptide hotspot by optimizing digestion times, buffer pH and using low pH-resistant proteases^13,23–25^. Collectively, these optimizations ensured a sensitive and reproducible methodology.

As proof of concept, the approach was employed to show concordance in data generated using similar assays in separate laboratories, and to evaluate immune responses in vaccine recipients against a panel of antigens. Care must be taken to apply the appropriate data normalizations to account for differences in assay dilution schemes when comparing between laboratories or across antigens. The variation in the slope and intercept of the resulting *MASCALE* conversion formulas indicate differences in sensitivity and background between each assay, possibly due to disparity in the epitope specificity of the reference standard antibodies related to each antigen, which can be affected by even subtle changes in protein sequence and folding. Major advantages of the method are that repeat calibrations are not needed, nor are changes in ELISA methodology used for sample analysis necessary.

*MASCALE* could address broader questions in the field of vaccine-induced immunity. It is often necessary or desirable to perform comparisons with greater degrees of complexity, incorporating multiple assays, different research laboratories and cross-study analysis, as exemplified by the SARS-COV-2 pandemic response^26,27^. The method could assist in establishing commonly accepted correlates of protection within a given disease area by defining an absolute threshold of binding antibodies needed for protection, allowing comparisons of data across various settings to advance scientific understanding and accelerate the development of interventions. Instead of requiring bridging studies to compare across disparate ELISA units which are difficult to interpret, this method could establish, for example, a required microgram level of antibody induced by vaccination. Correlating immune levels to those required for protection against an infectious disease, and applying that across variants would significantly accelerate vaccine development and optimization^28^.

Moving to a quantitative standard for antibody measurement allows comparison of antibody levels between diseases and between vaccines. This method can be adapted to other fields of common need, including biologics development and diagnostics, it is generalizable to any ELISA, provided a suitable representative peptide can be identified. It may also be applicable to studying subclass- and subtype-specific responses, which are of interest given the identification of subclass-specific responses as correlates of protection in previous HIV vaccine trials, as well as for profiling the diversity of individual response to infection or vaccination^29,30^. A further application is bridging of immune responses between species, for example, to provide insight into the translation of preclinical results in non-human primates to clinical results in humans^31^. This would avoid the need to develop assays with common detection reagents that are often not available. *MASCALE* could be applied to evaluate the role and impact of pre-existing immunity, such as toward Influenza, to examine the impact on disease susceptibility or vaccine-induced immune responses, where pre-existing antibodies have been associated with differential response to vaccination^32^. Furthermore, the method can be used to determine a specific activity, such as neutralization or Fc-specific function per microgram of antibody, providing an indication of the potency of antibodies against specific antigens and pathogens.

While *MASCALE* could in theory be applied to any ELISA data set, we ensured that data for responses in HIV-1 vaccine recipients to a panel of antigens could be confidently merged and evaluated together by standardizing methodology and reference standard for the 17 ELISAs employed for analysis. Although only the target antigen varied, the interpretation of relative immune measurements was not the same when comparing arbitrary versus absolute concentrations. Where this alignment is not possible, the method has even greater value in allowing quantitative comparisons when larger differences between assays exist. As protocols for measurement of protein concentration have been defined^33^, so too can specific antibodies now be evaluated in absolute terms. The *MASCALE* method satisfies an unmet need for absolute quantitation of binding antibody responses in biological samples and has great potential to advance data analysis options and scientific understanding of (pre)clinical trial results and beyond.

## Methods

### Target peptide calibration by mass spectrometry

Universal surrogate peptides representative of human IgG1, 3, 4 (VVSVLTVLHQDWLNGK; MW 1808.09 g/mol) and human IgG2 (VVSVLTVVHQDWLNGK; MW 1794.06 g/mol)^13^, as well as the stable isotope-labeled peptides used as internal standards (VVSVLTVLHQDWLNGK* and VVSVLTVVHQDWLNGK*; K* = Lys ^13^C_6_, ^15^N_2_) were synthesized at JPT Peptide Technologies. Peptides unique to human IgG and present in the Fc region were selected based on favorable characteristics for mass spectrometric analysis, including optimal length (6–20 amino acids), absence of N-linked glycans, adequate chromatographic retention, efficient ionization, and suitable chromatographic peak shape. Stock solutions were prepared in water/acetonitrile/formic acid (70:20:10; v/v/v) and stored at −20 °C. A calibration curve of each peptide was generated from 0.4 to 1000 pg peptide/sample for further quantitation of total IgG amounts in clinical samples via mass spectrometric analysis. QC samples consisting of known amounts of peptide (2.5 pg/sample, 25 pg/sample, and 500 pg/sample run in duplicate) were prepared in sample buffer (digested bovine serum albumin (BSA; Pierce)). Run acceptance criteria were set based on ICH M10 and Bioanalytical Method Validation Guidance for Industry^10,34^. An analytical run was accepted if at least 4 out of 6 of the QC results and at least 1 of the 2 QC replicates at each concentration level were within 20% of their nominal value.

### Method qualification for conversion formula generation

The mass spectrometry method to determine peptide calibration curves was qualified by assessment of accuracy, precision, selectivity, dilution integrity, robustness, and reagent stability using peptides for hIgG1,3,4 and hIgG2 (Supplementary Table 1a). To qualify the sample preparation and method process to obtain the slope and intercept for the conversion formula, a human IgG1 monoclonal antibody specific to HIV-1 Env was used to create a reference standard curve to assess assay precision (repeatability and intermediate precision) and accuracy (Supplementary Table 1b).

### Reference standard sample preparation

To create a reference curve in absolute mass units for quantitation of antigen-bound total IgG antibodies, the reference standard curve bound to coated antigen was prepared for each ELISA using the same method applied for clinical sample analysis. The reference standard points assigned values from 100,000 EU/mL were bound to HIV-1 Env antigen mimicking precisely the assay conditions used for the ELISA. Following a wash step to remove unbound antibodies and serum components, samples were denatured overnight in 8 M urea in 50 mM ammonium acetate, pH 5.5 (8 M urea buffer). 16 replicate wells were pooled per point on the standard curve into LoBind 96-well plates to enable downstream analysis, and each standard curve was constructed in triplicate for mass spectrometric measurements. BSA was added to each sample to a final concentration of 5 µg/mL to reduce non-specific binding. A known amount of purified human IgG (α-SARS-CoV-2) was included as a control to evaluate and adjust for digestion efficiency, and ELISA assay buffer was included for background correction.

### Filter-assisted sample preparation

Denatured samples were applied to pre-wet Vivacon Hydrosart^®^ stabilized cellulose filters (30KDa molecular weight cutoff; Sartorius) connected to LoBind tubes for reduction and alkylation steps. Samples were reduced with 20 mM dithiothreitol (DTT; Sigma) in 8 M Urea buffer for 30 min at 37 °C, followed by alkylation of free cysteines with 50 mM iodoacetamide (IAA; Sigma) in 8 M urea buffer for 20 min at room temperature (RT) in the dark. The flowthrough was discarded, and filters were washed three times with 2 M urea in 50 mM ammonium acetate, pH 5.5 (2 M urea buffer) before digestion for 3 h at 37°C using rLys-C and trypsin from the AccuMAP^TM^ Low pH Protein Digestion Kit (Promega). Tubes were subsequently centrifuged to collect tryptic peptides in the flowthrough. 20% acetonitrile (Biosolve) in 50 mM ammonium acetate pH 5.5 was added to the filter to reduce non-specific binding followed by a further centrifugation step. Finally, 1% acetic acid in LC-MS grade water (Biosolve) was added to the flowthrough to inactivate enzymes prior to storage of the sample at −80 °C until solid-phase extraction and analysis by LC-MS/MS.

### Solid-phase extraction

Samples were subjected to reversed-phase solid-phase extraction using Sep-Pak tC18 96-well plates (40 mg Sorbent; Waters Corporation), pre-washed with acetonitrile to activate the resin, followed by 0.6% acetic acid in water. Samples were spiked with stable isotope-labeled peptides used as an internal reference, applied to the Sep-Pak tC18 96-well plates, and briefly centrifuged. QC samples consisting of known amounts of (non-labeled) peptide spiked into digested BSA were included for analysis alongside clinical samples. Wells were washed twice by addition of 0.6% acetic acid in water and further centrifugation. Samples were then eluted with 0.6% acetic acid in acetonitrile/water (80:20; v/v) and evaporated under nitrogen at 35 °C for ~3 h until dry. Samples were then reconstituted in water/acetonitrile/formic acid (70:20:10; v/v/v).

### Liquid chromatography with tandem mass spectrometry (LC-MS/MS)

Chromatographic separation was carried out on a LC-30AD system (Shimadzu) with a 2.1 × 50 mm (1.7 µm) Acquity Ultra Performance Liquid Chromatography (UPLC) BEH300 C18 column (Waters Corporation) at a flow rate of 0.4 mL/min using a LC-30 ACMP autosampler (Shimadzu Nexera). Mobile Phase A (MP A) consisted of 0.1% formic acid in water; Mobile Phase B (MP B) consisted of 0.1% formic acid in acetonitrile. Samples were separated by gradient elution starting at 10 % MP B, followed by linear increase over 5 min to 40% MP B, followed by a step gradient to 98% MP B, which was held for 1 min before returning to the initial start conditions.

The liquid chromatography system was coupled to a QTRAP 6500+ Triple Quad mass spectrometer (Sciex) operating in the multiple reaction monitoring (MRM) positive ionization mode. Generic parameters were as follows: curtain gas 35 psi; ionization voltage (IS) 5000 V; source gas 1 (GS1) 70 psi and source gas 2 (GS2) 40 psi; collision activated dissociation (CAD) gas 9 psi; declustering potential (DP) 50 V and entrance potential (EP) 10 V. The peptide-specific parameters are provided in Supplementary Table 6. Stable isotope-labeled peptide signal was used to compensate for recovery variation from solid-phase extraction and matrix effects in the mass spectrometry ionization phase. Peak Area Ratios for light/heavy isotopes were plotted against the peptide concentration, and linear regression was performed with 1/x^2^ weighting to obtain the peptide calibration curves. Quantities were obtained in pg peptide (representative of IgG1,3,4 and IgG2) per sample by back-calculation of the peak area ratio onto the peptide calibration curve. Analyst software v1.7 was used for LC-MS/MS acquisition. Data processing was performed using SciexOS v2.1.6 software.

### Conversion formula generation

The amount of each surrogate peptide measured by LC-MS/MS was used to determine a formula for the conversion of arbitrary EU/mL values to absolute quantities of IgG per well. Analysis was performed using JMP software (SAS). Values below the quantifiable range of the ELISA were excluded from the analysis. Variability of replicate measurements was calculated, and 1 data point was excluded if %CV was >15%. Data were corrected for digestion efficiency as determined from % recovery of the digestion control (hIgG), and further corrected for the number of wells used for the measurement to obtain the pg mass of each target peptide per well (Supplementary Fig. 6). The amount of each peptide measured was then converted to moles of peptide, followed by conversion to the mass of IgG1, 3, 4 and IgG2, then summed to obtain the total amount of IgG per well. The mass of IgG per well was log_10_ transformed for plotting against the corresponding log_10_ EU/mL value assigned to the reference standard sample. A conversion formula in the form y = ax + b was generated following fitting by linear regression to obtain slope and intercept values.

### HIV-1 Env panel selection

A panel of HIV Env sequences from clade B and C was selected to assess cross-clade binding antibody responses. Clade B sequences were selected from the acute strains recorded in the Los Alamos National Laboratory (LANL) database. Clade C sequences were selected from a representative panel of acute clade C viruses in Southern Africa^35^. Strain selection was performed randomly and subsequently verified to guarantee sequence diversity and exclude closely related strains by plotting each strain on the phylogenetic tree of the corresponding clade. Reference strains were included based on prior knowledge of expression profile. Distribution of antigenic properties such as loop length, electrostatic charge and glycosylation sites were later checked against overall clade properties. A total of 11 clade B and 6 clade C strains formed the panel.

### HIV-1 Env panel design, production, and purification

The panel of 17 HIV-1 Env sequences was engineered to include a minimum number of consensus repair^18^ and structure-based stabilization mutations (A501C, T605C, I559P^19^; L556P, K655I, K658V^18^) to enable expression and uniform purification of trimeric proteins in prefusion conformation, while preserving diversity of viral attributes and epitope integrity.

The HIV-1 Env gp140 proteins were produced in HEK293E cells and purified with Galanthus nivalis lectin beads followed by size-exclusion chromatography (SEC) as previously described^18^. Fractions containing the gp140 trimer were pooled and stored for further analysis. Purity and mass were confirmed by size-exclusion chromatography and multi-angle light scattering (SEC-MALS) using a high-performance liquid chromatography (HPLC) system (Agilent Technologies) and microDAWN instrument coupled to a microT-rEX Refractive Index Detector (Wyatt). In total, 10 µg of purified protein was applied to a Unix-C SEC-300 column, coupled to a corresponding guard column (Sepax). The data were analyzed using Astra 6 software, and molecular weight was derived from the refractive index signal.

### HIV-1 Env characterization by monoclonal antibody and serum panel

Purified HIV-1 Env proteins were assessed by ELISA for binding to a panel of monoclonal antibodies and commercially sourced sera from HIV-infected individuals (BioIVT). The serum panel assessed was obtained from both male and female donors, varying in age, race, and ethnicity (Supplementary Table 7). Monoclonal antibodies used for evaluation were produced as human IgG using published sequences of the variable domains for the following antibodies with varying specificities: *V2-apex/trimer specific*: PGT145^36^, PGM1400^37^, CAP256-VRC26.25^37^; *V3-base*: PGT128^36^, 2G12^38^; *V3 crown*: 14E^39,40^, 447-52D^41^; *CD4-binding site*: B6^42^, B12^43^, F105^44^; *gp120/gp41 interface*: PGT151^42^, 35O22^44^; *CD4-induced*: 17b^45^.

### ELISA for HIV-1 Env characterization

HIV-1 Env was directly coated onto 96-well half-area high-binding white plates (Perkin Elmer) at 1 µg/mL. Wells were washed with 0.05% Tween-20 in PBS (wash buffer) and blocked in 1% casein in PBS (block buffer). Plates were subsequently incubated with 2.5-fold serial dilutions of monoclonal antibody or sera from HIV-infected individuals (from 6 µg/mL or 20× dilution, respectively). Wells were washed prior to incubation with 0.2 µg/mL horse radish peroxidase (HRP) conjugated goat-α-human IgG (Jackson ImmunoResearch). After a final wash, enhanced chemiluminescent (ECL) substrate (BioRad) was added before the development of a luminescence signal, which was measured in an Envision plate reader (Perkin Elmer).

For improved curve fitting, an offset of 10,000 was added to raw data values prior to log_10_ transformation and fitting of data using a 4-parameter logistic regression model with no restrictions on the top, slope and EC_50_, and a fixed bottom value. Endpoint titers were calculated if values were above a cutoff of 1.5 times the geometric mean of the low control (buffer only).

### Assay qualification and validation for HIV-1 Env ELISA clinical sample testing

To qualify the ELISA for the HIV-1 Env panel prior to clinical sample analysis, the following parameters were assessed at Pharmaceutical Product Development (PPD, Inc.): linearity, assay precision (repeatability and intermediate precision), relative accuracy, quantifiable range (lower limit of quantification; LLOQ to upper limit of quantification; ULOQ), dilutional linearity and specificity.

Vaccine-homologous HIV-1 Env C97ZA assays were validated at both Janssen and PPD, Inc., and included in addition to the qualification parameters, assessment of the assay cut-point, selectivity, interference, and sample and reagent stability (Supplementary Tables 2 and 3).

### Analysis of clinical samples for binding antibody responses to HIV-1 Env

Serum samples evaluated by ELISA at PPD, Inc. and Janssen for vaccine-homologous (Clade C C97ZA) responses against HIV-1 Env were selected from 4 clinical studies to assess comparability between laboratories. Samples were selected to include results spanning the assay range and were obtained from the APPROACH^46^ [ClinicalTrials.gov Identifier: NCT02315703], TRAVERSE^47^ [NCT02788045], ASCENT^20^ [NCT02935686] and IMBOKODO [NCT03060629] studies. The studies were conducted in accordance with the Declaration of Helsinki, Good Clinical Practices, and applicable regulatory guidelines. Study protocols were approved by institutional review boards at each study site, as detailed on each clinicaltrials.gov listing.

Serum samples for assessment of immune responses to the HIV-1 Env panel selected for sequence diversity in Clade B and C were obtained from the ASCENT Phase 1/2a study to assess the safety and immunogenicity of an investigational HIV-1 prophylactic vaccine regimen. The study regimen assessed consisted of Ad26.Mos4.HIV administered intramuscularly at day 0 and week 12, followed by Ad26.Mos4.HIV co-administered with clade C gp140 and Mosaic gp140 (Mos1) at weeks 24 and 48. The regimen tested is the same regimen evaluated for efficacy in the MOSAICO study (ClinicalTrials.gov identifier NCT03964415). Samples collected 4 weeks following completion of the primary vaccination series (week 52), were assessed by ELISA for vaccine-induced responses.

The ELISA was performed as previously described^46^. In brief, serum samples were added to antigen-coated and blocked 96-well microtiter plates at the required assay dilution. HIV-1 Env-specific antibodies were detected with a HRP conjugated mouse anti-human IgG antibody (Jackson ImmunoResearch), followed by the development of a colorimetric reaction using tetramethylbenzidine (TMB) substrate, before measurement of optical density at 450 nm. Each plate contained a 12-point standard reference, as well as high, medium, low, and negative quality control serum samples to guarantee assay validity. The samples, the reference standard, and the controls were measured in duplicate. Four-parameter logistic (4PL) regression was used to fit the reference curve where the highest concentration of the standard reference was arbitrarily set at 100,000 ELISA units per mL (EU/mL). EU/mL concentrations were determined per sample by back-calculation of the measured OD 450 value onto the reference curve. Samples returning results above the quantifiable range of the assay were re-tested following further pre-dilution to obtain a reportable value.

### Analysis of quality control samples for binding antibody responses to RSV F protein

Quality Control (QC) serum samples to determine assay validity and monitor trending were assessed by ELISA for binding antibody responses to Respiratory Syncytial Virus (RSV) subtype A (strain A2) and subtype B (strain B17) prefusion (F) protein, as previously described^15^. To set QC acceptance ranges prior to clinical sample analysis, low (LQC), mid (MQC) and high (HQC) responders were selected per antigen from commercially acquired human serum from healthy individuals testing positive for RSV F-specific antibodies during screening. The mean QC response per antigen was determined from more than 60 replicates across multiple assays performed on different days. The QC acceptance range was derived using the 95% β-expectation tolerance interval for the sample when Type I error was fixed at 0.01 for the 2/3 rule (i.e., at least 2 out of 3 QC samples were required to pass the QC acceptance criteria to accept an assay plate). QC responses in arbitrary units were converted to absolute quantities of IgG per mL serum using the *MASCALE* method.

### Data analysis and statistical methods

Participant samples from baseline and post-vaccination were evaluated by ELISA. Participants were defined as responders if post-baseline values obtained were above LLOQ in those with baseline values < LLOQ. If baseline values were ≥LLOQ, participants were defined as responders if post-baseline values were threefold higher than baseline. If data at baseline was not available, the participant was defined as a responder if post-baseline values were >LLOQ. Source data from ELISA obtained in EU/mL were converted to pg IgG/mL using the conversion formula specific for each ELISA. Correction for assay dilution scheme and well volume was performed to obtain quantity values per mL of serum (Supplementary Fig. 6).

### Equivalence assessment

To compare results across ELISAs at separate laboratories, proportionality and systematic difference was assessed. To assess proportionality, Deming regression was performed on the log_10_ EU/mL or log_10_ pg IgG/mL concentration measured at one laboratory versus the other, using the appropriate variance ratio for each assay. To evaluate systematic difference between results generated at each laboratory, the difference in log_10_ EU/mL or log_10_ pg IgG/mL result is first calculated per sample. The average difference in log_10_ EU/mL or log_10_ pg IgG/mL is then calculated with a 90% confidence interval across samples.

### Magnitude-breadth analysis

A magnitude-breadth plot was provided to report arbitrary or absolute antibody concentrations for each individual serum sample assayed across a panel of antigens. Magnitude-breadth curves assess the proportion of antibody concentrations in the panel greater than a certain EU/mL or pg IgG/mL threshold. A clade-specific curve was included based on the average magnitude breadth across all participants in clade B and in clade C. A Wilcoxon log-rank test was applied to the area under the curve (AUC) for clade B and clade C and a related *P* value was reported. A 95% confidence interval was calculated for the fold difference between the AUCs (ratio Clade C/Clade B) using a paired *t* test. The correlation between clades was plotted, reporting the overall magnitude by participant, and an associated Pearson correlation coefficient was calculated.

### Reporting summary

Further information on research design is available in the Nature Research Reporting Summary linked to this article.

### Supplementary information

Supplementary Materials

REPORTING SUMMARY

## Data Availability

Janssen has an agreement with the Yale Open Data Access (YODA) Project to serve as the independent review panel for the evaluation of requests for clinical study reports and participant-level data from investigators and physicians for scientific research that will advance medical knowledge and public health. Data will be made available following publication and approval by YODA of any formal requests with a defined analysis plan. For more information on this process or to make a request, please visit The YODA Project site. The data sharing policy of Janssen (Pharmaceutical Companies of Johnson & Johnson) is available online.
