# Supplementary material for: Absolute quantitation of binding antibodies from clinical samples
Source: NPJ Vaccines. 2024 Jan 6;9:8. doi: 10.1038/s41541-023-00793-w (PMC10771429; doi:10.1038/s41541-023-00793-w)
Supplement: Supplementary file 1 — Supplementary Materials [file 41541_2023_793_MOESM1_ESM.pdf]

Supplementary Material

Absolute Quantitation of Binding Antibodies from Clinical Samples

Chan Tang[1*], Annemiek Verwilligen[1*], Jerald Sadoff[1], Boerries Brandenburg[1], Eveline Sneekes-Vriese[1], Tom van den Kerkhof[1], Lieve Dillen[2], Lucy Rutten[1], Jarek Juraszek[1], Katleen Callewaert[2], Sarah Janssen[1], Jeroen Huizingh[1], Zelda Euler[1], Tom Schilperoord[1], Marc Verhemeldonck[2], Johannes PM Langedijk[1], Jenny Hendriks[1], Daniel J Stieh[1, a]

* These authors contributed equally to this work.

[a] Corresponding author: Daniel J. Stieh, Telephone: +1 272 253 1576, E-mail: dstieh@vax.co. Current address: Vaccine Company, Inc., South San Francisco, United States.

Affiliations

1. Janssen Vaccines & Prevention, Leiden, The Netherlands
2. Janssen Research & Development, Beerse, Belgium

Supplementary Table 1 – Method Qualification Parameters for Conversion Formula Slope and Intercept

Qualification of the surrogate peptide calibration method by mass spectrometry was performed to assess accuracy, precision, selectivity, dilution integrity, robustness, and reagent stability using surrogate peptides for hIgG1,3,4 and hIgG2. Results are shown in Table 1a.

Parameter	Acceptance Criteria	Human IgG1, 3, 4 surrogate peptide	Human IgG2 surrogate peptide
Calibration range (pg/sample)	LLOQ: RE < 25%<br>ULOQ: RE <20%	LLOQ: 0.400 pg/sample RE -3.5%<br>ULOQ: 1000 pg/sample RE 2%	LLOQ: 0.400 pg/sample RE -3%<br>ULOQ: 1000 pg/sample RE -2.5%
Accuracy and precision (within run)	3 concentration levels (N=6/level)<br>RE< 20%; CV < 20%	LQC (2.5 pg/sample): RE 9%; CV 3.4%<br>MQC (25.0 pg/sample): RE 6.5%; CV 8.5%<br>HQC (500 pg/sample): RE 8.1%; CV 4.3%	LQC (2.5 pg/sample): RE 4.4%; CV 4.0%<br>MQC (25.0 pg/sample): RE 8%; CV 9.9%<br>HQC (500 pg/sample): RE 8.2%; CV 3.6%
Dilution Linearity 10x	RE < 20%; CV< 20% (n=6)	SHQC (5000 pg/sample): RE 1%; CV 4.9%	SHQC (5000 pg/sample): RE 4.3%; CV 4.8%
Selectivity (6 different sources)	Response (PA) analyte < 25% of LLOQ<br>Response (PA) IS < 5% of LLOQ	Analyte: 3 out of 6 sources PA>25% of PA LLOQ*<br>IS 6 out of 6 sources PA< 5 % of PA in LLOQ	Analyte: 2 out of 6 sources PA>25% of PA LLOQ*<br>IS 6 out of 6 sources PA< 5 % of PA in LLOQ
Stability stock solution<br>3 days RT<br>4 F/T	RE < 20% versus reference<br>(N=3 per condition)	2500 pg/mL<br>3 days RT, RE 3%<br>4 F/T, RE 5%	2500 pg/mL<br>3 days RT, RE -3.7%<br>4 F/T, RE -2.1%
Stability in matrix<br>24h RT	RE < 20%<br>CV < 20%<br>N=3	MQC (25.0 pg/sample): RE -8.5%; CV 3.2%	MQC (25.0 pg/sample): RE -9.9%; CV 2.4%

Supplementary Table 1a. Qualification characteristics of VVSVLTVLHQDWLNGK (representative of human IgG1, 3, 4) and VVSVLTVVHQDWLNGK (representative of human IgG2) surrogate peptide calibration by LC-MS/MS. Accuracy and precision measurements were performed using peptide QC samples prepared at 3 concentrations in digested BSA solution. 3 concentration levels were assessed (N=6 replicates per level and analyte) in one qualification experiment. RT: Room temperature; F/T: freeze-thaw; LLOQ: Lower limit of quantification; ULOQ: Upper Limit of Quantification; IS: Internal Standard; RE: Relative error; PA: Peak area; CV: Coefficient of Variation; N: number of samples; LQC: Low Quality Control; MQC: Mid Quality Control; HQC: High Quality Control, SHQC: Super High Quality Control.

* Selectivity evaluation did not meet pre-specified acceptance criteria, therefore, in each analytical run, selectivity was evaluated and the LLOQ raised to meet the Selectivity acceptance criteria if needed.

Performance parameters for the method process including sample preparation steps were determined by incubation of known amounts of purified IgG1 to HIV-1 Env antigen coated ELISA plates to create a reference standard curve. All steps of the method were executed to obtain the conversion formula to relate assigned input values to absolute quantities of IgG per sample. Two different operators performed an experiment in triplicate on three separate days. In each experiment, linear regression curves (N=3) were constructed for measured amount of surrogate peptide from IgG1 (representative of IgG1, 3 and 4) versus the input amount. The overall repeatability (N=9) and intermediate precision (average of the N=3 individual experiments) was determined on the slope of the individual linear regression curves. Relative accuracy was determined for each of the IgG1

concentration levels evaluated. The IgG1 concentrations with consistent performance (>LLOQ) across all 9 calibration curves ranged from 35 ng to 143 pg/sample. Data analysis was performed in JMP version 14.2, and the results are shown in Table 1b.

	**Repeatability**	**Intermediate precision**
Slope	4.76 % (CV)	6.59% (CV)
Accuracy (for the concentration range 143 pg-35 ng)	76-120%	

Supplementary Table 1b. Qualification parameters for method process to determine conversion formula, including sample preparation, mass spectrometry and linear regression. CV: Coefficient of Variation.

Supplementary Table 2 – ELISA Validation Parameters for HIV-1 Env C97ZA Antigen at Laboratory 1 (Janssen)

Assay Parameter	Acceptance criteria	Result for Antigen: HIV-1 Env C97ZA	Status
Linearity and Precision	LLOQ and ULOQ: CV IP <25% + TE <40%<br>90% CI Slope within 0.8 – 1.25<br>% CV Overall Precision <30%	156.25 – 5,000 (EU/mL)<br>0.968 – 1.000<br>13.6%	Pass
Dilutional Linearity 1x – 250x	90% CI Slope within 0.8 – 1.25<br>% CV IP each dilution < 20%	Dilution: 1, 5, 10, 20, 40, 80, 100, 150, 200 and 250<br>1.12 – 1.15	Pass
Cut-point (95th percentile HIV negative commercial samples)	NA	O.D.450: 0.44	NA
Assay range	NA	156.25 – 1,250,000 EU/mL	NA
Specificity	Heterologous recovery within [80% - 120%]<br>Homologous recovery [<25%]	110%<br>6%	Pass
Interference	GMR and<br>90% CI within [-0.15 $\log_{10}$; 0.15 $\log_{10}$]	Hemolytic: -0.054 – 0.015<br>Lipemic: 0.016 – 0.084<br>Icteric: 0.007 – 0.076	Pass
Sample freeze-thaw stability (Univariate analysis + post-hoc Dunnett's t-test)	GMR and<br>90% CI within [-0.15 $\log_{10}$; 0.15 $\log_{10}$]	9 F/T: -0.030 – 0.059<br>12 F/T: -0.037 – 0.052	Pass

Supplementary Table 2. ELISA validation parameters and outcome for HIV-1 Env C97ZA antigen performed at Laboratory 1 (Janssen). NA: Not applicable; GMR: Geometric mean ratio; CI: Confidence interval; CV: Coefficient of variation; IP: Intermediate precision; TE: Total Error; EU/mL: ELISA units per milliliter; F/T: freeze-thaw.

Supplementary Table 3 – ELISA Validation Parameters for HIV-1 Env C97ZA Antigen at Laboratory 2 (PPD, Inc).

Assay Parameter	Acceptance Criteria	Result for Antigen: HIV-1 Env C97ZA	Status
LLOQ (EU/mL)	NA	80	NA
ULOQ (EU/mL)	NA	14720	NA
Intermediate Precision (%CV IP)	≤ 30%	13.2%	Pass
Linearity (Slope and 90% CI)	90% CI within [0.80, 1.25]	1.029 (1.024, 1.034)	Pass
Intermediate Precision Clinical Trial Samples (%CV IP)	% CV ≤ 30%	Neat: 18.7%<br>1:10: 10.8%	Pass
Dilutional Linearity (Slope and 90% CI)*	90% CI within [0.80, 1.25]	0.99 (0.97, 1.01)	Pass
Cut-point (95th percentile negative clinical samples)	NA	168 EU/mL	NA
Assay range	NA	80 – 1,884,160 EU/mL	NA
Specificity (Heterologous antigen) (GMR and 90% CI)	90% CI within [0.70, 1.43]	1.00 (0.95, 1.05)	Pass
Selectivity (GMR and 90% CI)	90% CI within [0.70, 1.43]	1.10 (1.06, 1.14)	Pass
Interference (GMR and Dunnett corrected 90% CI)	90% CI within [0.70, 1.43]	Hemolytic:1.00 (0.94, 1.05)<br>Lipemic: 0.92 (0.87, 0.97)<br>Icteric: 0.96 (0.90, 1.02)	Pass
Sample freeze-thaw stability (GMR and Dunnett corrected 90% CI)	90% CI within [0.70, 1.43]	24hr at RT: 0.97 – 1.00<br>15 F/T: 0.97 – 1.03	Pass

* Maximum pre-dilution 1:128.

Supplementary Table 3. ELISA validation parameters and outcome for HIV-1 Env C97ZA antigen performed at Laboratory 2 (PPD Inc.,). NA: Not applicable; GMR: Geometric mean ratio; CI: Confidence interval; CV: Coefficient of variation; IP: Intermediate precision; EU/mL: ELISA units per milliliter; F/T: freeze-thaw.

.

Supplementary Table 4 – Peptide Calibration Curve Data

Run ID	ELISA and Laboratory for sample analysis	Peptide	Calibration equation (y= ax + b)	R^2	Weighting
1	Clade C Laboratory 1	hIgG1, 3, 4	y = 0.08824x + 0.00737	0.99879	$1/x^2$
		hIgG2	y = 0.09217x + 0.00910	0.99466	$1/x^2$
2	Clade C Laboratory 2	hIgG1, 3, 4	y = 0.08782x + 0.04288	0.9923	$1/x^2$
		hIgG2	y = 0.08968x + 0.00551	0.99356	$1/x^2$
3	Clade C Laboratory 1	hIgG1, 3, 4	y = 0.08841x - 0.00954	0.99878	$1/x^2$
		hIgG2	y = 0.08989x - 0.00883	0.99705	$1/x^2$
4	Clade C Laboratory 2	hIgG1, 3, 4	y = 0.08417x + 0.01246	0.99813	$1/x^2$
		hIgG2	y = 0.08850x - 0.00664	0.99904	$1/x^2$

Supplementary Table 4. Peptide calibration curve equations for *MASCALE* quantitation of HIV-1 Env C97ZA ELISA responses at Laboratory 1 and Laboratory 2 obtained per run. x = peptide concentration; y = peak area ratio of peptide (light/heavy isotope); a = slope; b = intercept.

Supplementary Table 5 – ELISA Qualification Parameters for HIV-1 Env Panel

Table 5A	HIV-1 Env Antigen (Clade B)											Outcome	
**Parameter**	**136172**	**607523**	**635056****	**APV_13**	**APV_14**	**APV_17**	**APV_18**	**CNE10**	**ES_X2515**	**REJO_67**	**SC422.8**	**Criteria**	**Status**
LLOQ (EU/mL)	203	202	299	588	189	200	456	633	429	198	786	-	-
ULOQ (EU/mL)	52032	103360	85040	46744	97504	51280	263680	112112	78880	50560	112032	-	-
Intermediate Precision (%CV IP)	8.8%	11.0%	21.1%	17.2%	10.3%	13.3%	8.6%	9.4%	10.8%	7.6%	9.4%	≤ 30%	Pass
Linearity (Slope and 90% CI)	0.978 (0.969, 0.986)	0.981 (0.972, 0.991)	1.017 (1.009, 1.025)	0.955 (0.946, 0.963)	0.991 (0.982, 1.000)	0.982 (0.972, 0.991)	0.949 (0.937, 0.961)	0.937 (0.929, 0.946)	0.989 (0.975, 1.004)	0.979 (0.973, 0.985)	0.911 (0.900, 0.922)	90% CI within [0.80, 1.25]	Pass
Intermediate Precision Clinical Samples (%CV IP)	11.2%	16.8%	24.8%	16.2%	14.9%	14.7%	19.7%	25.7%	16.2%	9.6%	14.3%	≤ 30%	Pass
Dilutional Linearity (Slope and 90% CI)*	0.88 (0.86, 0.90)	0.87 (0.84, 0.90)	0.91 (0.89 0.93)	0.90 (0.88, 0.92)	0.88 (0.86, 0.90)	0.90 (0.87, 0.92)	0.84 (0.82, 0.86)	0.89 (0.86, 0.91)	0.84 (0.83, 0.86)	0.98 (0.95, 1.00)	0.90 (0.89, 0.92)	90% CI within [0.80, 1.25]	Pass
Specificity (Heterologous antigen) (GMR and 90% CI)	1.03 (0.98, 1.07)	1.03 (0.88, 1.20)	1.10 (1.01, 1.20)	1.03 (0.91, 1.16)	1.03 (0.94, 1.13)	1.03 (0.94, 1.15)	1.05 (1.00, 1.11)	0.99 (0.96, 1.03)	0.97 (0.89, 1.06)	1.04 (0.99, 1.09)	0.98 (0.94, 1.04)	90% CI within [0.70, 1.43]	Pass

* Maximum pre-dilution: 1:2048.

** Acceptance criteria for antigen 635056 were not met for dilutional linearity when tested at 1:8 and 1:32 dilution.

Table 5B	HIV-1 Env Antigen (Clade C)						Outcome	
**Parameter**	**20915593**	**16055-2.3**	**21492713 _B11_E3**	**CNE30**	**DU422.01* ***	**TRP343_2 _00_21_2**	**Criteria**	**Status**
LLOQ (EU/mL)	182	440	211	386	213	410	-	-
ULOQ (EU/mL)	93120	124368	108832	98880	108992	121664	-	-
Intermediate Precision (%CV IP)	8.2%	11.5%	7.6%	9.3%	8.6%	8.7%	≤ 30%	Pass
Linearity (Slope and 90% CI)	1.001 (0.991, 1.011)	0.963 (0.955, 0.970)	0.966 (0.955, 0.977)	1.042 (1.032, 1.053)	1.004 (0.997, 1.011)	1.025 (1.014, 1.035)	90% CI within [0.80, 1.25]	Pass
Intermediate Precision Clinical Samples (%CV IP)	12.9%	27.7%	9.0%	13.9%	11.0%	13.0%	≤ 30%	Pass
Dilutional Linearity (Slope and 90% CI)*	0.87 (0.84, 0.89)	0.88 (0.86, 0.89)	0.96 (0.94, 0.98)	0.95 (0.93, 0.97)	1.04 (1.01, 1.07)	0.91 (0.89, 0.93)	90% CI within [0.80, 1.25]	Pass
Specificity (Heterologous antigen) (GMR and 90% CI)	1.04 (0.99, 1.09)	1.05 (1.01, 1.10)	1.15 (0.94, 1.40)	1.02 (0.99, 1.06)	1.05 (1.01, 1.10)	1.04 (0.98, 1.11)	90% CI within [0.70, 1.43]	Pass

* Maximum pre-dilution: 1:2048.

** Acceptance criteria for DU422.01 were not met for dilutional linearity when tested at 1:128 dilution.

Supplementary Table 5a and 5b. ELISA qualification parameters and results for the HIV-1 Env ELISA panel for Clade B (Table **5a**) and Clade C (Table **5b**) antigens showing acceptance criteria and status. NA: Not applicable; GMR: Geometric Mean Ratio; CI: Confidence Interval; CV: Coefficient of Variation; IP: Intermediate Precision; EU/mL: ELISA Units per milliliter.

Supplementary Table 6 – Mass Spectrometry Parameters Applied for Target Peptides

Peptide	Signature Peptide Sequence	Molecular weight (g/mol)	Q1 Mass (m/z)	Q3 Mass (m/z)	Dwell (ms)	CE (eV)	CXP (V)
hIgG1,3,4	VVSVLTVLHQDWLNGK	1808.09	603.6	805.7	35	23	32
hIgG2	VVSVLTVVHQDWLNGK	1794.06	598.9	798.7	35	23	10
Stable isotope labelled hIgG1,3,4	VVSVLTVLHQDWLNG**K*** K*= Lys $^{13}C_6$, $^{15}N_2$	1816.03	606.3	809.7	35	23	32
Stable isotope labelled hIgG2	VVSVLTVVHQDWLNG**K*** K*= Lys $^{13}C_6$, $^{15}N_2$	1802.00	601.6	802.7	35	23	10

Supplementary Table 6. Mass spectrometry parameters applied for target peptides VVSVLTVLHQDWLNGK (representative of human IgG1, 3, 4) and VVSVLTVVHQDWLNGK (representative of human IgG2). Q1: Quadrupole 1; Q3: Quadrupole 3; Dwell: Dwell time; m/z: mass to charge; ms: millisecond; CE collision energy; eV: excitation voltage; CXP: collision exit potential; V: voltage.

Supplementary Table 7 – Serum Panel used for HIV-1 Env Antigen Panel Characterization

Name	Vendor	Source	Gender	Age (Yrs)	Race	Ethnicity	Diagnosis	Medications
HMN341960 (Serum #1)	BioIVT	Human serum; HIV+	Male	4	Northern Mandes	Northern Mandes	HIV, Pediatric Donor	ABC-3TC-NVP; ABC-3TC-LPV/r
HMN341961 (Serum #2)	BioIVT	Human serum; HIV+	Female	5	Krous or Southern Mandes	Krous or Southern Mandes	HIV, Pediatric Donor	AZT-3TC-LPV/r; ABC-3TC-LPV/r
HMN341963 (Serum #3)	BioIVT	Human serum; HIV+	Female	46	Krous or Southern Mandes	Krous or Southern Mandes	HIV 2	None
HMN341966 (Serum #4)	BioIVT	Human serum; HIV+	Male	50	African	African American	HIV, Normal Donor	None
HMN341968 (Serum #5)	BioIVT	Human serum; HIV+	Male	61	Akan	Akan	HIV-2	None
HMN341969 (Serum #6)	BioIVT	Human serum; HIV+	Female	45	Akan	Akan	HIV-2	None
HMN341970 (Serum #7)	BioIVT	Human serum; HIV+	Female	49	Burkina Faso (Gouroussi)	Burkina Faso (Gouroussi)	HIV-2	None
HMN341971 (Serum #8)	BioIVT	Human serum; HIV+	Male	69	Krous or Southern Mandes	Krous or Southern Mandes	HIV-2	None
STA-0054-00050 (Serum #9)	BioIVT	Human serum; HIV+	Male	39	Black	Not available	Not available	Not available

Supplementary Table 7. Serum panel used for HIV-1 Env antigen panel characterization. Panel consists of commercially sourced sera from HIV-infected individuals. Available donor data are shown.

Supplementary Figure 1

1	ASTKGPSVFP	LAPSSKSTSG	GTAALGCLVK	DYFPEPVTVS	WNSGALTSGV
51	HTFPAVLQSS	GLYSLSSVVT	VPSSSLGTQT	YICNVNHKPS	NTKVDKKVEP
101	KSCDKTHTCP	PCPAPELLGG	PSVFLFPPKP	KDTLMISRTP	EVTCVVVDVS
151	HEDPEVKFNW	YVDGVEVHNA	KTKPREEQYN	STYRVVSVLT	VLHQDWLNGK
201	EYKCKVSNKA	LPAPIEKTIS	KAKGQPREPQ	VYTLPPSRDE	LTKNQVSLTC
251	LVKGFYPSDI	AVEWESNGQP	ENNYKTTPPV	LDSDGSFFLY	SKLTVDKSRW
301	QQGNVFSCSV	MHEALHNHYT	QKSLSLSPGK		

Supplementary Fig. 1a. Human immunoglobulin heavy chain gamma 1 (IgG1) amino acid sequence (Uniprot P01857) with the selected surrogate peptide for human IgG1,3 and 4 highlighted in red.

1	ASTKGPSVFP	LAPCSRSTSE	STAALGCLVK	DYFPEPVTVS	WNSGALTSGV
51	HTFPAVLQSS	GLYSLSSVVT	VPSSNFGTQT	YTCNVDHKPS	NTKVDKTVER
101	KCCVECPPCP	APPVAGPSVF	LFPPKPKDTL	MISRTPEVTC	VVVDVSHEDP
151	EVQFNWYVDG	VEVHNAKTKP	REEQFNSTFR	VVSVLTVVHQ	DWLNGKEYKC
201	KVSNKGLPAP	IEKTISKTKG	QPREPQVYTL	PPSREEMTKN	QVSLTCLVKG
251	FYPSDISVEW	ESNGQPENNY	KTTPPMLDSD	GSFFLYSKLT	VDKSRWQQGN
301	VFSCSVMHE	ALHNHYTQKS	LSLSPGK		

Supplementary Fig 1b. Human immunoglobulin heavy chain gamma 2 (IgG2) amino acid sequence (Uniprot P01859). The selected surrogate peptide for human IgG2 is highlighted in red. The amino acid highlighted in blue shows the difference in sequence from the surrogate peptide used for human IgG1,3 and 4 (shown in Fig. 1a).

Supplementary Figure 2

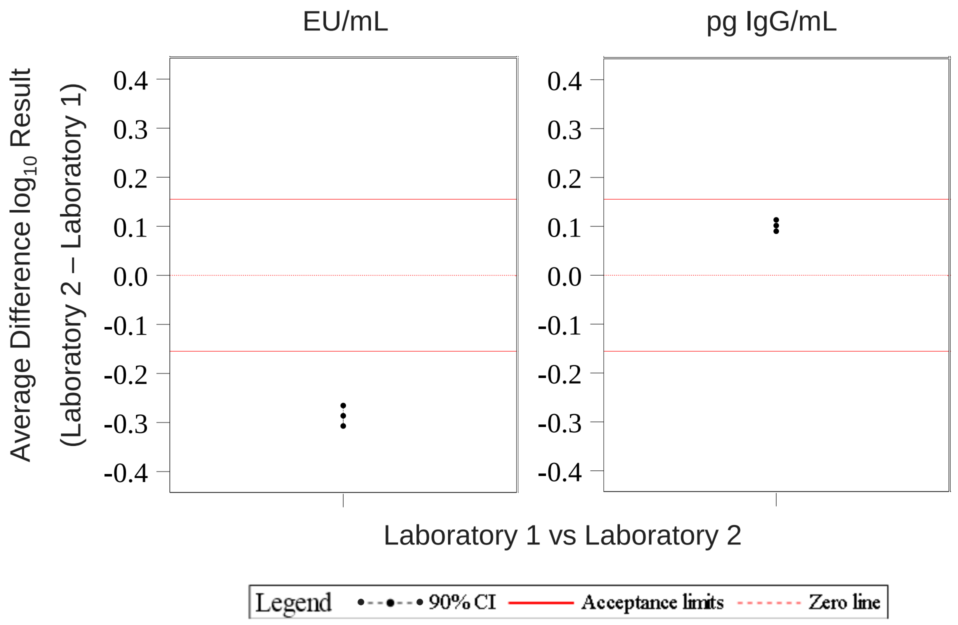

ELISA Readout	Antigen	Average Difference log10 Result	Alpha	Lower limit 90% CI	Upper limit 90% CI	90% CI within [-0.155 log10, 0.155 log 10]
log 10 EU/mL	HIV-1 Env gp140	-0.286	0.1	-0.307	-0.265	No
log10 pg IgG/mL	HIV-1 Env gp140	0.102	0.1	0.090	0.113	Yes

Supplementary Figure 2. Equivalence assessment for ELISA responses to vaccine-matched clade C (C97ZA) antigen measured at two laboratories (Laboratory 1 and Laboratory 2). The average difference of the log10 transformed EU/mL or pg IgG/mL values between laboratory 2 and laboratory 1 is indicated with 90% confidence interval (CI). Red lines indicate pre-specified equivalence limits of -0.155 log10; 0.155 log10. The dotted red line indicates the zero-difference line.

Supplementary Figure 3

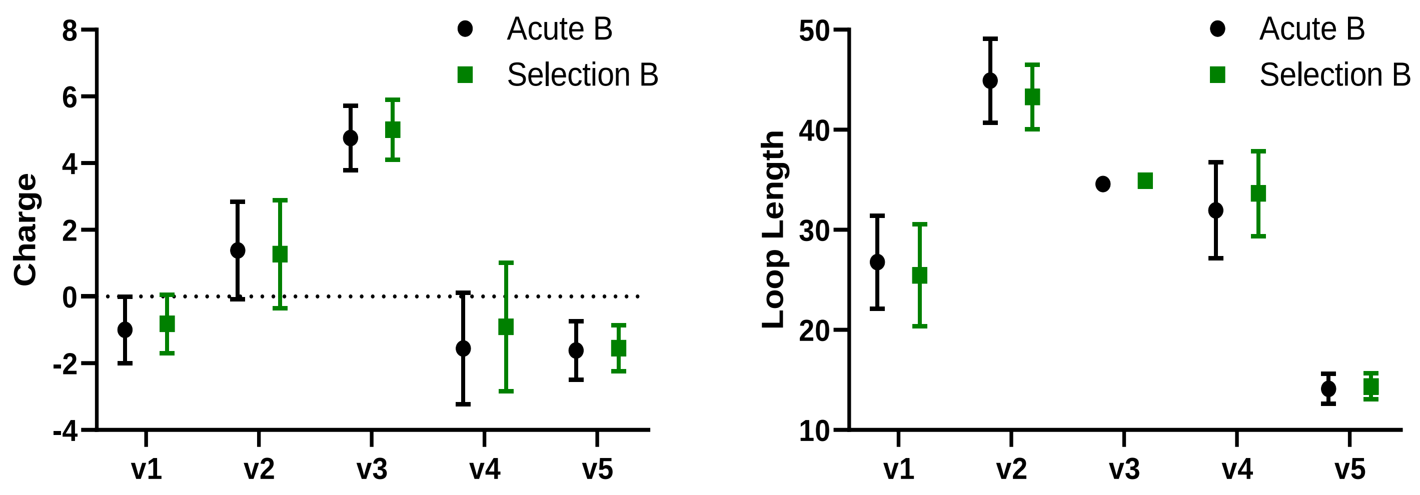

Glycosylation Sites

	N130	N156	N160	N262	N295	N332	N334	N442	N448
Acute Clade B	74%	100%	83%	98%	85%	87%	9%	2%	91%
Selection Clade B	73%	100%	82%	100%	91%	91%	9%	0%	91%
APV_18	1	1	0	1	1	1	0	0	1
CNE10	1	1	1	1	1	1	0	0	1
APV_14	1	1	1	1	1	1	0	0	1
REJO_67	0	1	1	1	1	0	1	0	1
635056	0	1	1	1	1	1	0	0	0
SC422.8	1	1	1	1	1	1	0	0	1
ES_X2515_3	1	1	1	1	1	1	0	0	1
APV_17	1	1	1	1	0	1	0	0	1
136172	1	1	1	1	1	1	0	0	1
607523	1	1	0	1	1	1	0	0	1
APV_13	0	1	1	1	1	1	0	0	1

STRAIN	V1 SEQUENCE	V2 SEQUENCE	V4 SEQUENCE	V5 SEQUENCE
APV_18	CTDVGDATNTKNRSKGEMEKGEIKNC	CSFNVATRISNRMQKEYALFYKLDVVPIDNVNTTEKKNNYTKYRLISC	CNTTQLFNSTWRSNDTTQLSNSTWRSNDTASEGSNDTIILPC	RDGGNNSTNNTEVF
CNE10	CSNLNTTNTSSTIEGGEIKNC	CSFNITTSISTKVKDYAIFNKLDIVPIGNDTTSYILINC	CNTSQLFNSTWNVTGTVNGTTGNGTITLPC	RDGGNESTTTETETF
APV_14	CTDDLTNTTFRNSTTFENSSLIEKGEIKNC	CSFNVTTILRDKVHKEHALFYKLDVVPIDDNNTTNTSYRLINC	CNTTLLFDSTWNNNSTWNANVSETIILPC	RDGGIGNSNSENETF
REJO_67	CTDLNVTNSNSTDHSTNSSLEAKGEIKNC	CSFNITTTPRDKIQKEYAIFYKQDVVPIKNDNISYRLISC	CNTAQLFNSSWDTNTNGNDTQGPSENNTIILPC	RDGGNSSLSSPEIF
635056	CTDLNVTNTNSSSAKNTNNSMIDGGEMKNC	CSFNVTTNLRDKMEREFALLYKLDIVPIGNDSNSNSYRLISC	CDTTQLFNSNSTWLFNNTWNSTENNNTENNNITLQC	RDGGSSDQNNTEIF
SC422.8	CTDELRNGTYANVTVTEKGEIKNC	CSFNITTAIRDKVQKTYALFYRLDVVPIDNNHGNSSSNYSNYRLINC	CNTTQLFNSTWNINGTWHGTTVSNKTIILPC	RDGGNNNSTTEIF
ES_X2515_3	CSPYYTNVTNNTNNNTTVANTNNSSGLEGITGELRNC	CSFNVTEEINKKMRKEYAIFNEIDIVKISNESYMLKNC	CNTSKLFNSTLFSNNTWFNSTMNETNTNDTIMIPC	RDGGGGQNQTENQTEIF
APV_17	CTEVKNNASDPKQDKEVTNC	CSFNITTSIRDKVQKEYAIFYKYDVVPIDDSDNNSTNTSYRLISC	CNTSKLFNSTWHANGTRINGTNVYANGTNATNTIITLPC	RDGGNNTNGSEIF
136172	CTDYQGNGTNTNRTNTNSSIEGDGEIKNC	CSFNYTTNIRDKIQRTYALFYKLDVVPIEKNSSSYRLISC	CDTTKLFNSTWNSTWNNTTRNDTGGEDKITLPC	RDGGNTSSTIETF
607523	CTDYRGNVTITNSSSSSEVKNC	CSFDITTSINEKVKGSALFYKYDVIPINNNNDNTSYRLLSC	CNTSRLFNSTWNTTDIQNYTELNTTERENITLQC	RDGGGNGTGNNETF
APV_13	CHNVMNKTNMTSEKEVIGEMKNC	CSFNVTTNIRDKVKSEYALFYKLDIVQIKEDGSNTSYRLTSC	CNTTQLFNGTWNSTWSSTVENGTITLPC	RDGGNNNNTANETEIF

Supplementary Figure 3. HIV-1 Env Clade B panel characteristics compared to acute clade B strains in LANL database. Diversity in amino acid sequence (bottom), charge (top left), loop length (top right) and glycosylation (middle) is shown for the selected clade B strains in the panel.

Supplementary Figure 4

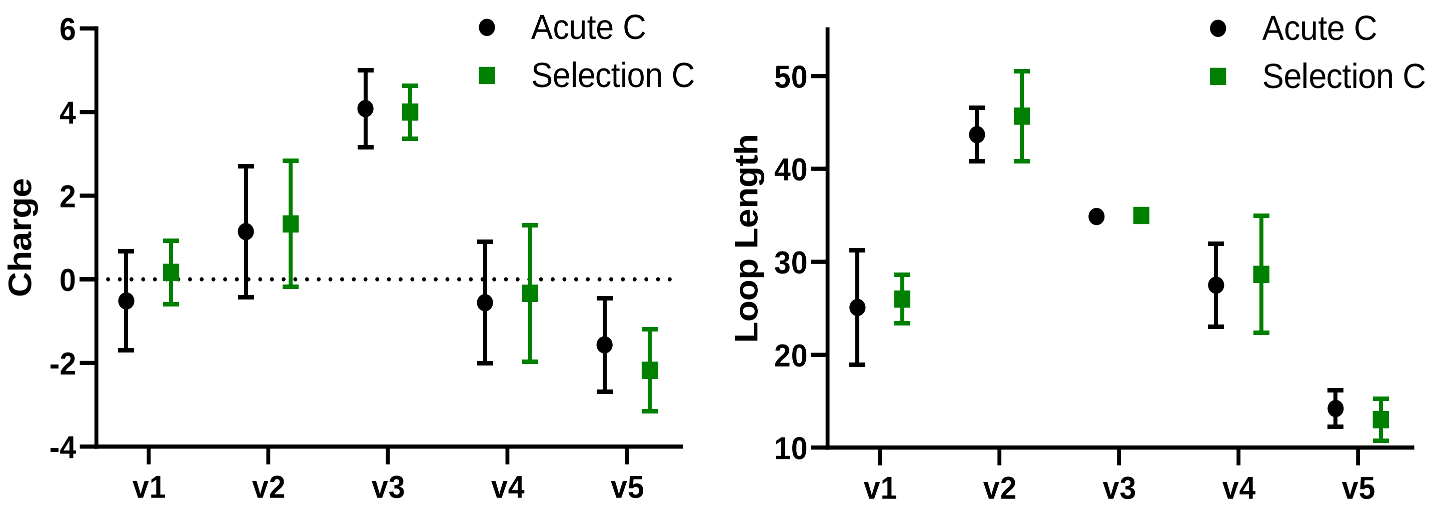

Glycosylation Sites

	N130	N156	N160	N262	N295	N332	N334	N442	N448
Acute Clade C	49%	96%	90%	100%	23%	70%	15%	77%	70%
Selection Clade C	33%	100%	83%	100%	33%	83%	0%	83%	83%
DU422.01	0	1	1	1	0	1	0	1	1
21492713_B11_E3	1	1	0	1	0	1	0	1	1
CNE30	0	1	1	1	0	1	0	1	1
20915593	1	1	1	1	1	1	0	1	0
TRP343_2_00_21_2	0	1	1	1	1	1	0	0	1
16055-2.3	0	1	1	1	0	0	0	1	1

STRAIN	V1 SEQUENCE	V2 SEQUENCE	V4 SEQUENCE	V5 SEQUENCE
DU422.01	CKNVNISANANATATLNSSMNGEIKNC	CSFNTTTELRDKKQKVYALFYKPDVVPLNGGEHNETGEYILINC	CDTTKLFNETKLFNESEYVDNKTIILPC	WDGGENSTEGVF
21492713_B11_E3	CSNVNVTTSNITNSANNSNIRGGQEMKNC	CSFNVHRVLKDKKEKEYALFYKLDIVQLNSNSNSSEYRLINC	CNTSRLFNRNSTESKSTSNSTITLPC	RDGGMDNNNTEEIF
CNE30	CGNVSVSSNGTVASNKTYVEEMRNC	CSFNTTTFISDKHKKEHALFYRLDIVPLDDENSNKNCSKNSGQNCSEYYRLINC	CNTSDLFNSTYMSNGTFRFNGTVFNGTGGDSSINITIPC	RDGGTTNNTNNTEIF
20915593	CTANITLANVTLSGSSGNITGEMRNC	CSFNTTTEVRDKKKKEYALFYRLDIIPLTNESNESSGEYRLINC	CNTSKLFNGTYNGTGNATITLPC	HDGGENETF
TRP343_2_00_21_2	CVDVNSTTSSNNTTANNATANNSIKGEEKNC	CSFNTTTEIRDKEKKVNAIFYRSDIVPLNENSSDYRLINC	CNTSKLFNETETKSNISNITLPC	RDGGGGNETNHTETF
16055-2.3	CRQVNTTNATSSVNVTNGEEIKNC	CSFNATTEIRDKKQKVYALFYRLDIVPLEEERKGNSSKYRLINC	CNTSSLFNSTYNPNDTNSNSSSSNSSLDITIPC	RDGGVESNETEIF

Supplementary Figure 4. HIV-1 Env Clade C panel characteristics compared to acute clade C strains in LANL database. Diversity in amino acid sequence (bottom), charge (top left), loop length (top right) and glycosylation (middle) is shown for the selected clade C strains in the panel.

Supplementary Figure 5

Strain	Clade	Mutations
SC422.8	B	A501C T605C I559P L556P K655I K658V V329A F384Y
CNE10	B	A501C T605C I559P L556P K655I K658V A36V I97K Q234N S419R A636D
APV_14	B	A501C T605C I559P L556P K655I K658V V95M
APV_17	B	A501C T605C I559P L556P K655I K658V I175L H291S A297T S302N A320T A321G R564H G585R R629M
REJO_67	B	A501C T605C I559P L556P K655I K658V R16W Q63T I65V S98N I175L A254V A312G M583V S585R R629M
APV_13	B	A501C T605C I559P L556P K655I K658V G30A I65V Q564H M593L
607523	B	A501C T605C I559P L556P K655I K658V I226L V285I I454L L470P V491I
136172	B	A501C T605C I559P L556P K655I K658V L104M E238P I377N L513V R592L
ES_X2515	B	A501C T605C I559P L556P K655I K658V I122L S241N S266A V443I Q629M
APV_18	B	A501C T605C I559P L556P K655I K658V I42V L225I V272I D554N
635056	B	A501C T605C I559P L556P K655I K658V Q61Y Y93F Q448N A534S
16055-2.3	C	A501C T605C I559P L556P K655I K658V
CNE30	C	A501C T605C I559P L556P K655I K658V F164E S166R L225I H320T
21492713_B11_E3	C	A501C T605C I559P L556P K655I K658V
TRP343_2_00_21_2	C	A501C T605C I559P L556P K655I K658V S98N T471G T629M G647E
20915593	C	A501C T605C I559P L556P K655I K658V W210F A341T I448N
DU422.01	C	A501C T605C I559P L556P K655I K658V D33N G466E F643Y

Stabilizing mutations (SOSIP) Stabilizing mutations (RnS) Repair mutations

Supplementary Figure 5. HIV-1 Env antigen panel design showing consensus repair (black; Rutten *et al*.,[16]) and stabilizing mutations (blue; Sanders *et al*.,[19] and pink; Rutten *et al*., [18]) included per strain. Mutations were engineered to ensure uniform production and folding of antigens for downstream use in ELISA evaluations.

Supplementary Figure 6

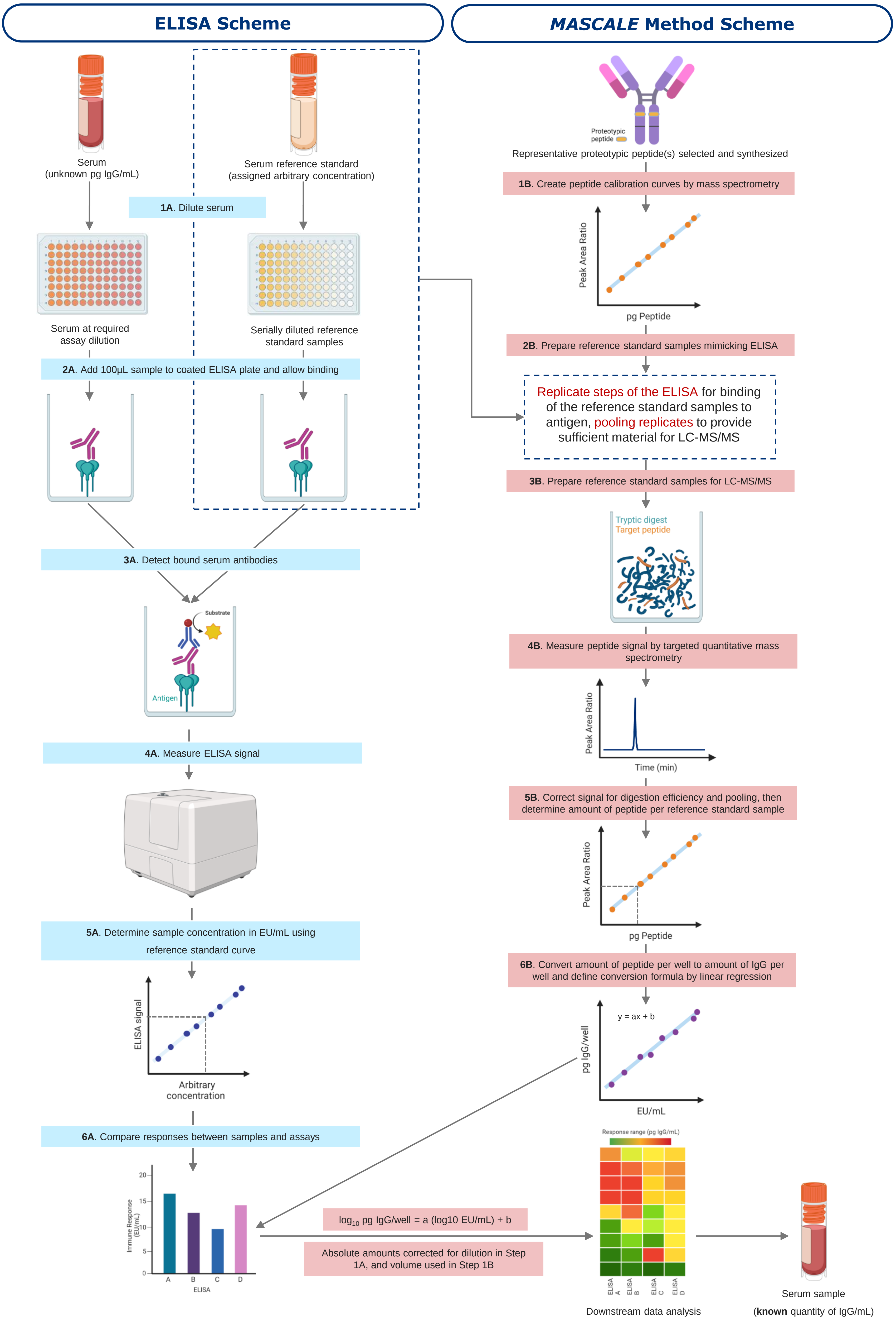

Supplementary Figure 6. ELISA and *MASCALE* Scheme with Data Processing Steps. A) ELISA Scheme. Serum is diluted to the required dilution for ELISA. Serial dilutions of the assay reference standard are prepared. 100µL of diluted sample is added to coated antigen on the ELISA plate and allowed to bind. After a wash step, bound antibodies are detected following incubation with HRP-conjugated secondary antibody and addition of substrate. The ELISA signal is measured in a plate reader and arbitrary concentrations determined using the reference standard curve. Responses can be compared between samples and assays. **B) *MASCALE* Scheme.** Representative peptides for human IgG are selected and synthesized. A peptide calibration curve is constructed by mass spectrometry. ELISA reference standard samples bound to antigen are prepared for mass spectrometry mimicking precisely steps of the respective ELISA. Replicate reference standard curves are prepared for pooling of samples and replicate measurements by mass spectrometry. Washed samples are processed by denaturation, reduction, alkylation and tryptic digestion, prior to being subjected to targeted quantitative mass spectrometry on a triple quad mass spectrometer. The MS signal is corrected for digestion efficiency as determined from % recovery of the digestion control (hIgG), and further corrected for the number of wells used for the measurement to obtain the per well mass of each target peptide. The mass of each peptide measured (IgG1,3,4 and hIgG2) is converted to moles, followed by conversion to mass of IgG, then summed to obtain the total amount of IgG/well. The mass of IgG/well is $\log_{10}$ transformed for plotting against the corresponding $\log_{10}$ EU/mL value assigned to the reference standard sample. A conversion formula in the form y = ax + b is generated by applying linear regression to obtain slope and intercept values. The conversion formula is applied to ELISA data in EU/mL to obtain the pg IgG/well. Values are corrected for sample dilution and volume used in the ELISA, obtaining results in pg IgG/mL serum.
